# Binankadsurin A from *Kadsura coccinea* Fruits Ameliorates Acetaminophen-Induced Liver Injury Through Inhibiting Oxidative Stress by Keap1/Nrf2/HO-1 Pathway

**DOI:** 10.3390/nu18030403

**Published:** 2026-01-26

**Authors:** Guy Paulin M. Kemayou, Yashi Wang, Muhammad Aamer, Chuanle Li, Shiqi Liu, Huanghe Yu, Caiyun Peng, Simeon F. Kouam, Bin Li, Wei Wang, Yupei Yang

**Affiliations:** 1 TCM and Ethnomedicine Innovation & Development International Laboratory, School of Pharmacy, Hunan University of Chinese Medicine, Changsha 410208, China; guybeni93@gmail.com (G.P.M.K.); 18173724730@163.com (Y.W.); m.aamer196267@gmail.com (M.A.); 14794431027@139.com (C.L.); shiqiliu670@163.com (S.L.); yhh@hnucm.edu.cn (H.Y.); caiyunpeng@hnucm.edu.cn (C.P.); libin@hnucm.edu.cn (B.L.); 2 Department of Chemistry, Higher Teacher Training College, University of Yaounde I, Yaounde P.O. Box 47, Cameroon; kfogue@yahoo.com

**Keywords:** *Kadsura coccinea*, binankadsurin A, acetaminophen, oxidative stress, liver injury, hepatoprotective

## Abstract

Objectives: *Kadsura coccinea* fruit is a traditional medicinal plant rich in dibenzocyclooctadiene lignans, with established hepatoprotective effects. Binankadsurin A (BKA), a dibenzocyclooctadiene lignan isolated from the *K. coccinea* fruits. This study aims to evaluate its hepatoprotective efficacy in an acetaminophen (APAP)-induced mouse liver injury model. Methods: The structure of BKA was elucidated by HR-ESI-MS, NMR, single-crystal X-ray diffraction and comparison of their data with those of the literature. Mice were randomly divided into five groups: Control, APAP (400 mg/kg, single intraperitoneal injection), APAP + bicyclol (50 mg/kg), APAP + low-dose BKA (50 mg/kg), and APAP + high-dose BKA (100 mg/kg). Untargeted metabolomics, immunohistochemistry, Western blot analysis, and molecular docking were performed. Results: BKA was determined as a dibenzocyclooctadiene lignan, and the single-crystal structure is reported for the first time. The untargeted metabolomics revealed that metabolites and pathways are closely associated with oxidative stress. In vivo studies showed that pretreatment with BKA can mitigate liver injury. BKA reduced serum levels of aspartate aminotransferase (AST) and alanine aminotransferase (ALT) and stored hepatic glutathione (GSH) levels. Immunohistochemical analysis results also showed that CYP2E1 expression in the mouse liver could be improved through BKA pretreatment. Furthermore, Western blot analysis presented that BKA could increase the protein expression of Nrf2, HO-1, and NQO-1. Additionally, molecular docking indicated that BKA directly blocks the binding site of Nrf2 with Keap1. Conclusions: BKA reduces APAP-induced acute liver damage by inhibiting oxidative stress by activating the Keap1/Nrf2/HO-1 signaling pathway, providing a theoretical basis for BKA as a potential therapeutic agent for APAP-induced liver injury.

## 1. Introduction

Acetaminophen (APAP), an antipyretic and analgesic drug, is among the most commonly used drugs for treating pain and fever [1]. Excessive or long-term use of APAP often triggers inflammatory reactions and oxidative stress, leading to drug-induced liver injury and even acute liver failure [2]. Currently, the most effective antidote for APAP overdose is N-acetylcysteine (NAC), but its narrow therapeutic window, low bioavailability, and poor stability limit its clinical use [3,4]. Therefore, it is necessary to develop and find new drugs or treatments against APAP-induced liver injury.

The overdose of APAP may cause significant oxidative damage in the liver. About 10% of APAP is metabolized by the cytochrome P450 enzyme system (CYP), generating the toxic metabolite *N*-acetyl-*p*-benzoquinone imine (NAPQI) [5]. The cytochrome P450 2E1 (CYP2E1) subtype is the primary catalytic enzyme in this process. When glutathione (GSH) is depleted, NAPQI accumulates in liver tissue. It covalently binds to mitochondrial proteins, producing excess reactive oxygen species (ROS) [6,7]. Thus, the anti-oxidative stress pathway is the key to alleviating liver injury. Nuclear factor E2-related factor 2 (Nrf2) signaling is an important antioxidant pathway. Nrf2 acts as a transcription factor. It binds to antioxidant response elements (ARE), which are needed for expression of key antioxidant enzymes [8,9], including heme oxygenase-1 (HO-1) and NAD (P) H quinone oxidoreductase 1 (NQO-1). Activation of Nrf2 can lead to significant antioxidant responses, protecting cells from oxidative stress [10]. Previous studies show Nrf2 activity is primarily regulated by Kelch-like ECH-associated protein 1 (Keap1). Normally, Nrf2 binds Keap1 and remains in the cell. Upon stimulation, modifications to keap1cysteine residues cause conformational changes that release Nrf2 from the Keap1-Nrf2 complex. For instance, the natural product artemether exerts protective effects against acetaminophen-induced liver injury through activation of the Nrf2 signaling pathway [5]. Similarly, silybin demonstrates therapeutic efficacy in ameliorating acute liver failure by modulating the AKT/GSK3β/Nrf2/GPX4 signaling cascade [11]. These findings suggest that natural products have strong potential in the search for drugs to treat liver-related diseases.

Fruits of *Kadsura coccinea*, also known as Bufuna, are a dicotyledonous plant belonging to the Schisandraceae family and the *Kadsura* genus. Notably, the shape of the *K. coccinea* fruit is similar to that of a pineapple and is non-toxic, making it edible raw and possessing a sweet and sour taste. Furthermore, the fruit flesh is rich in vitamins and minerals, making it a wild fruit with high potential for food and medicinal use [12]. Phytochemistry investigation has shown that *K. coccinea* fruits are rich in lignans and triterpenes [13,14]. Importantly, pharmacological studies have found that lignan compounds possess anti-APAP toxicity and anti-non-alcoholic fatty liver disease activities [15,16]. A dibenzocyclooctadiene lignan, Binankadsurin A (BKA), has been isolated from *K. coccinea* fruits. In the early stage, our team found that several dibenzocyclooctadiene lignans can alleviate APAP-induced liver toxicity [17], while the underlying specific mechanism remains unclear. The aim of this work was to systematically explore the molecular mechanism by which the dibenzocyclooctadiene lignan BKA mitigates APAP-induced acute liver injury (ALI) through in vivo experiments. In the present study, C57BL/6J mice were selected to establish the APAP-induced hepatic injury model. Through this experimental model, we hope to clarify the mechanism of BKA in regulating hepatic oxidative stress and Nrf2/Keap1 signaling pathway in APAP-induced ALI. We anticipate this study will impact research by providing novel insights into natural product-based therapeutic strategies for APAP-related liver injury, and further inform healthcare practice by offering a potential candidate compound for alleviating both acute and chronic APAP-induced liver damage.

## 2. Materials and Methods

### 2.1. Equipment and Reagents

1D and 2D NMR spectra were obtained using a Bruker AV-600 spectrometer (600 MHz for ^1^H, and 150 MHz for ^13^C, CDCl_3_). HRESI-MS analysis was performed on a Waters UHPLC-H-CLASS/XEVO G2-XS Q-TOF.

Normal Saline (Kelun Pharmaceutical, Anyang, China); Bicyclol (National Institutes for Food and Drug Control, Beijing, China, 99.7%); AST and ALT assay kits (Mindray Bio-Medical Electronics, Shenzhen, China); Acetaminophen (National Institutes for Food and Drug Control, Beijing, China); Gel preparation kit, 10% and 12% (Wansheng Haotian Biotechnology, Shanghai, China); SuperECL Chemiluminescent Kit (Ningbo Youcheng Biomedical Technology, Ningbo, China); BCA Protein Assay Kit (Elabscience, Shenzhen, China); Immobilon^®^-PSQ PVDF (Merck KGaA, Darmstadt, Germany); GSH assay kit (Nanjing Jiancheng Bioengineering Institute, Nanjing, China); anti-Nrf2 (A2117, ABclonal, Wuhan, China); anti-HO-1 (10701-1-AP, Proteintech Group, Inc., Wuhan, China); anti-NQO1 (67240-1-Ig, Proteintech Group, Inc., Wuhan, China).

### 2.2. Plant Material

Fresh fruits of *K. coccinea* were collected in Huaihua City, China, and authenticated by Prof. Wei Wang, School of Pharmacy, Hunan University of Chinese Medicine. The material was stored at TCM and Ethnomedicine Innovation & Development International Laboratory, School of Pharmacy, Hunan University of Chinese Medicine, Changsha, Hunan (voucher specimen of fruits: 20221001).

**Figure 1:**
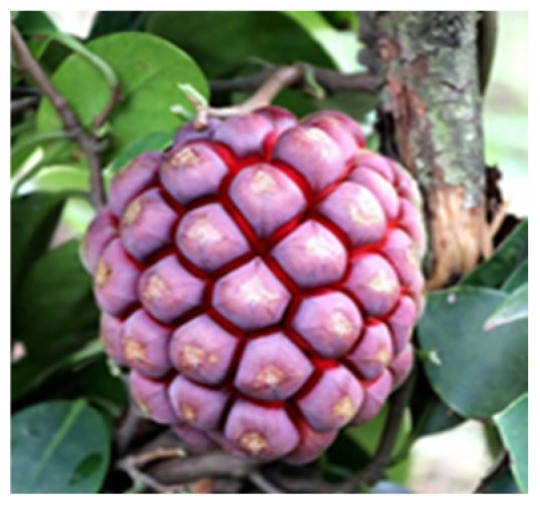
Picture of fresh fruit of *K. coccinea*.

The outer skin of *Kadsura coccinea* fruits is uniformly reddish-brown, with irregular wart-like protrusions (Figure 1). They taste sweet and sour, are slightly soluble in water (soluble in ethanol), and have a pH value of 4.2–4.8.

### 2.3. Extraction and Isolation

The fresh fruits of *K. coccinea* (20 kg) were extracted three times with 75% EtOH (3 × 200 L) at room temperature, then filtered [18]. The filtrate was evaporated under reduced pressure and partitioned with EtOAc (3 × 10 L). The EtOAc partition (119.1 g) was subjected to silica gel column chromatography using a PE–EA (1:0–0:1 gradient system) to obtain 12 fractions (A–L). Fraction E (12.4 g) was subjected to silica gel column chromatography using PE–EA (1:0–0:1) for elution to afford fractions E1–E7. Fraction E2 (741.4 mg) was separated on silica gel column chromatography, Sephadex LH-20 gel column chromatography, and recrystallization to obtain binankadsurin A (327 mg).

#### Binankadsurin A (BKA)

White amorphous powder; UV (MeOH) *λ_max_* 210 nm; IR (KBr) *ν_max_* 3396, 1699, 1652, 1558, 1506, and 1456 cm^−1^; HR-ESI-MS *m*/*z* 425.1571 [M + Na]^+^ (calcd. for C_22_H_26_O_7_Na, 425.1576). The UV purity of BKA, analyzed by HPLC, was more than 95%; ^1^H and ^13^C NMR data (Table 1); (Supporting Information, Figure S1). The BKA sample was isolated from the fresh fruits of *K. coccinea*.

### 2.4. Animal Model Establishment and Grouping

Male SPF-grade C57BL/6J mice (20–22 g) were purchased from Hunan Silaike Jingda Laboratory Animal Co., Ltd. (Animal Quality Certificate No.: 430727221101937162, Changsha, China). Animals were housed in barrier facilities at the Hunan Academy of Chinese Medicine. The housing environment fully complied with SPF-grade standards, with controlled conditions maintained (temperature: 20.0–26.0 °C; humidity: 40.0–70.0%). The mice were grouped at a density of five animals per polycarbonate cage with sterile bedding, and maintained under a 12 h light/dark cycle with free access to standard SPF-grade sterile chow and autoclaved drinking water. All mice were acclimatized to the housing environment for 3~5 consecutive days before the initiation of any experimental procedures to minimize stress-related variables.

Thirty male mice were randomly divided into five groups (*n* = 6 per group): (1) normal control, (2) APAP model, (3) positive control (bicyclol, 50 mg/kg), (4) low-dose BKA (50 mg/kg), and (5) high-dose BKA (100 mg/kg). After 3 days of acclimatization, all groups received intraperitoneal injections of their respective treatments twice at 24 h intervals. The normal and model groups received saline. Following a 12 h fasting period after the last administration, acute liver injury was induced in all groups except the normal control group, which was given a single intraperitoneal injection of APAP (400 mg/kg). Animals were sacrificed 24 h post-modeling for sample collection.

### 2.5. Serum Biochemical Parameters

Orbital blood was collected in an EP tube and stored at 4 °C for over 3 h. Once fully coagulated, the blood was centrifuged at 3000–3500 rpm for about 15 min to obtain serum. The serum AST and ALT levels were measured using the respective assay kits.

### 2.6. Measurement of Hepatic GSH Level

Tissue samples were weighed and homogenized in nine volumes of grinding medium. After centrifugation at 10,000–12,000 rpm for 10–15 min, the supernatant was collected to prepare a 10% tissue homogenate. The GSH levels were measured using a GSH assay kit.

### 2.7. HE Staining

Liver tissues were fixed in 4% paraformaldehyde, then processed through dehydration, clarification, paraffin infiltration, and embedding to prepare paraffin blocks. The paraffin blocks were sectioned into slices with a 5–6 μM thickness. The paraffin sections were baked at 65 °C for 2 h, dewaxed in a graded series of xylene and ethanol, stained with hematoxylin for 5–6 min, and rinsed with running water. The sections were then differentiated in acidified ethanol for 30 s and rinsed with running water. Subsequently, the sections were blued in 1% NaOH for 90–120 s and rinsed with running water. The sections were stained with eosin for 2 min and rinsed with running water. Afterwards, the sections were quickly dehydrated in 100%, 95%, 85%, and 75% ethanol for 5 s each, cleared in xylene I and II for 20 min each, and finally mounted with neutral gum. The results were analyzed under a light microscope.

### 2.8. Untargeted Metabolomics Study

Mouse liver tissues were weighed and placed into centrifuge tubes, to which 80% methanol aqueous extract (containing four internal standards) was added. The tubes were placed into a cryogenic grinding mill and ground for 2 min (60 Hz) at −20 °C. After cryogenic grinding, the tubes were kept at −20 °C for 30 min. The samples were next centrifuged at 4 °C for 15 min, and the resulting supernatant was transferred to vials for LC-MS analysis. The analysis was performed using an ultra-high-performance liquid chromatography coupled with Fourier transform mass spectrometry (UHPLC-Q Exactive HF-X) system from Thermo Fisher Scientific. Separation was achieved on an ACQUITY UPLC HSS T3 column (100 mm × 2.1 mm, 1.8 µm) under ESI+ and ESI− scanning modes with a mobile phase consisting of two components: solvent A (95% water and 5% acetonitrile with 0.1% formic acid) and solvent B (47.5% acetonitrile, 47.5% isopropanol, and 5% water with 0.1% formic acid). The injection volume was 3 µL, and the column temperature was maintained at 40 °C [19].

### 2.9. Immunohistochemistry Analysis

The paraffin-embedded tissue sections were baked at 65 °C for 2 h, then dewaxed using a graded series of xylene and ethanol. Next, the slides were immersed in preheated citrate buffer (pH 6.0) brought to a boil in a microwave oven. After boiling, the staining jar containing the slides was transferred to an insulation box and allowed to cool to room temperature. Once cooled, and after washing with PBS, endogenous peroxidase activity was blocked by incubating with peroxidase blocking reagent at room temperature for 10 min. Following another PBS wash, the CYP2E1 antibody was applied and incubated overnight at 4 °C. After the overnight incubation, a reaction enhancer solution was added and further incubated for 20 min at room temperature. For visualization, a freshly prepared DAB substrate was used for 1–2 min, followed by thorough rinsing with distilled water. Counterstaining was performed with hematoxylin for 20 s, differentiation for 30 s, and blueing for 90 s. After washing with distilled water, the sections were rapidly dehydrated through a graded ethanol series (75%, 85%, 95%, and 100%), with 5 s immersions at each concentration. The slides were then cleared in xylene I and II for 20 min each, and finally mounted with neutral balsam. Finally, the stained sections were examined and analyzed under a light microscope [20].

### 2.10. Western Blot Analysis

Subsequently, protein concentrations were quantified via the BCA assay. Samples were separated by SDS-PAGE (80 V for 30 min, and 120 V for 90 min) and transferred to PVDF membranes. After blocking with 5% skim milk, membranes were probed with primary antibodies against NQO1 (1:10,000), Nrf2 (1:1000), and HO-1 (1:5000) at 4 °C overnight. Secondary antibodies (1:10,000) were incubated for 2 h at room temperature. Signals were detected using an ECL substrate.

### 2.11. Molecular Docking

The 2D structure of BKA was first drawn using ChemDraw version 20.0. Next, the structure was energy-minimized using Chem 3D version 20.0. The PDB file of the keap1 protein was downloaded from the Protein Data Bank (PDB) at (https://www.rcsb.org/, PDB: 4IF1). The protein was then preprocessed using PyMOL version 2.5.0. The molecular docking was performed using AutoDock Tools version 1.5.6. Finally, the results were visualized and analyzed using PyMOL version 2.5.0 and LigPlus 2.2.0 version.

### 2.12. Statistical Analysis

Statistical analysis was performed using GraphPad Prism version 9.5.1. Shapiro–Wilk normality tests were first conducted to verify the distribution of all data sets, and the results indicated that all data conformed to a normal distribution (*p* > 0.05). Therefore, the data were expressed as the mean ± standard deviation (mean ± SD). The comparison among multiple independent groups was performed using the one-way ANOVA, followed by post hoc Tukey multiple comparisons test (*α* = 0.05) to determine whether there is a difference between the mean of all possible pairs, and false discovery rate is employed to adjust for multiple comparisons. * *p* < 0.05, ** *p* < 0.01, and *** *p* < 0.001.

## 3. Results

### 3.1. Structure Characterization of Binankadsurin A (BKA)

**Figure 2:**
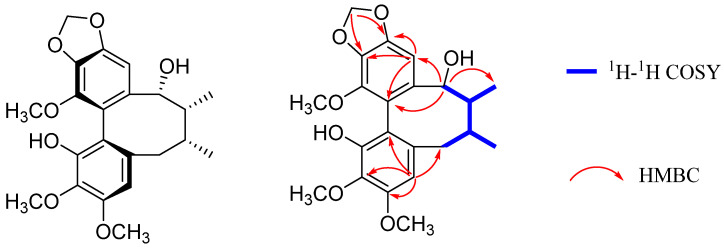
Chemical structure, Key ^1^H–^1^H COSY and HMBC correlations of BKA.

**Figure 3:**
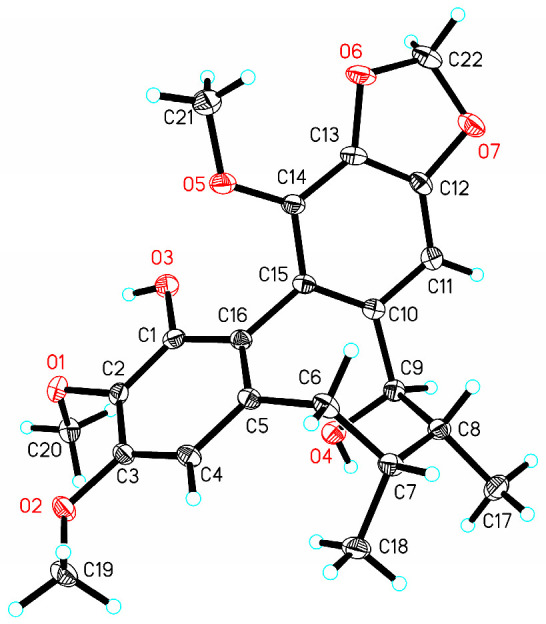
X-ray ORTEP drawing of BKA (Cu K*α*).

BKA was isolated as a white amorphous powder. Its molecular formula is evident from its HRESI-MS, which showed the pseudo-molecular peak [M + Na]^+^ at *m/z* 425.1571 (calc. for C_22_H_26_O_15_Na, 425.1576). This corresponds to ten degrees of unsaturation. The UV data showed an absorption maximum at λ_max_ 210 nm. Its IR spectrum showed absorption bands at 3396 (hydroxyl), 1506, and 1456 cm^−1^ (aromatic rings). These data were consistent with BKA being a dibenzocyclooctadiene lignan. The ^1^H NMR spectrum (Table 1) exhibited two aromatic singlets for a biphenyl moiety at *δ_H_* 6.41 (H-4) and 6.36 (H-11). Three methoxy groups singlets appeared at *δ_H_* 3.90 (H_3_-3), 3.88 (H_3_-14), and 3.87 (H_3_-2), with two singlets characteristic of a methylenedioxy group at *δ_H_* 5.97 and 5.95. A cyclooctadiene ring was recognized from two secondary methyl doublets at *δ_H_* 0.93 (H_3_-17, d, *J* = 7.4 Hz) and 1.16 (H_3_-18, d, *J* = 7.2 Hz), two methine at *δ_H_* 2.07 (H-7) and 1.91 (H-8), an oxymethine at *δ_H_* 4.63 (H-9), and a methylene at *δ_H_* 2.63 and 2.62 (H_2_-6). This was confirmed by ^1^H-^1^H COSY correlations between H-6/H-7/H-8/H-9 (Figure 2). The ^13^C NMR spectrum and DEPT-135° indicated 12 aromatic carbons at *δ_C_* 147.3, 133.8, 151.3, 107.4, 133.8, 138.8, 102.8, 148.9, 135.7, 141.4, 118.3, and 115.1. One methylenedioxy group appeared at *δ_C_* 101.2, three methoxy groups at *δ_C_* (59.8, 61.1, and 55.7), and two methyl groups at *δ_C_* (19.7 and 15.4). The HMBC spectrum showed protons of the methylenedioxy group (OCH_2_O) are correlated with C-12 (*δ_C_* 148.9) and C-13 (*δ_C_* 135.7). Thus, it was inferred that OCH_2_O was located at C-12 and C-13; H-4 correlates with C-1 (*δ_C_* 147.3), C-6 (*δ_C_* 38.8), and C-16 (*δ_C_* 115.1). H-11 correlates with C-9 (*δ_C_* 83.6), C-10 (*δ_C_* 138.8), C-12 (*δ_C_* 148.9), and C-15 (*δ_C_* 118.3). Therefore, the hydroxyl groups are assigned to C-1 and C-9. The NMR data were similar to binankadsurin A [14]. Thus, BKA was elucidated as shown (Figure 2). A single-crystal X-ray diffraction study of BKA (Figure 3) was successfully carried out. The results suggested that BKA possesses an *S*-biphenyl configuration, and the data revealed absolute configurations of C-7(*R*), C-8(*R*), and C-9(*R*). X-ray Crystallographic Analysis: (M = 402.43 g/mol): monoclinic, space group C2 (no. 5), a = 23.5810(6) Å, b = 7.9995(2) Å, c = 10.2215(3) Å, *β* = 95.926(3), V = 1917.84(9) Å3, Z = 4, T = 169.99(10) K, *μ* (Cu K*α*) = 0.860 mm^−1^, Dcalc = 1.394 g/cm^3^. A total of 5897 reflections were measured (7.538° ≤ 2Θ ≤ 147.548°), with 3425 unique (Rint = 0.0198, Rsigma = 0.0245) used in all calculations. The final R1 was 0.0308 [I > 2*σ*(I)], and wR2 was 0.0849 (all data). The Flack/Hooft parameter was 0.04(8)/0.05(6). Crystallographic data for BKA have been deposited at the Cambridge Crystallographic Data Center (CCDC: 2467421).

### 3.2. BKA Alleviates APAP-Induced ALI

**Figure 4:**
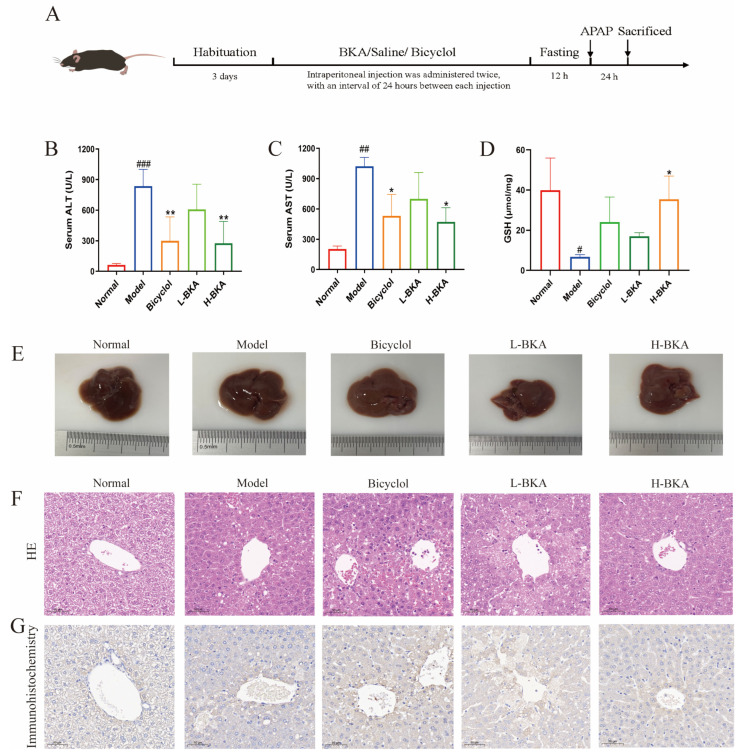
BKA attenuates APAP-induced liver injury in mice. An experimental process diagram (**A**). Serum ALT level (**B**). Serum AST level (**C**). Effects of ALI on liver GSH levels (**D**). The liver morphology of mice in different groups (**E**). HE staining section at 100× magnification (**F**). Representative images of CYP2E1 immunohistochemical analysis in the liver (**G**). Data are shown as the mean ± SEM, ^#^ *p* < 0.05 vs. control; ^##^ *p* < 0.01 vs. control; ^###^ *p* < 0.001 vs. control; * *p* < 0.05 vs. APAP; ** *p* < 0.01 vs. APAP.

A murine model of ALD was established by administering a single intraperitoneal injection of acetaminophen (APAP) at a dose of 400 mg/kg (Figure 4A). Compared to the control group, the APAP-treated group exhibited a significant increase in serum levels of ALT (*p* < 0.001) and AST (*p* < 0.01), which were reduced after treatment with H-BKA and bicyclol (Figure 4B,C). The hepatic tissue GSH levels were markedly decreased in the APAP group (*p* < 0.05). This reduction was significantly reversed by H-BKA treatment (Figure 4D). Additionally, the livers of the APAP group mice displayed a dark brown coloration and rough texture, indicative of inflammation or fibrosis, which were notably reversed by BKA treatment (Figure 4E). HE staining revealed pronounced hepatocyte necrosis, disordered cellular arrangement, nuclear dissolution, and prominent vacuolization in the cytoplasm of the APAP group compared to normal controls. Treatment with bicyclol and BKA significantly ameliorated these pathological changes, with 100 mg/kg BKA demonstrating superior efficacy compared to 50 mg/kg BKA (Figure 4F). The CYP2E1 expression level was evaluated in the mouse liver using immunohistochemical analysis. The results showed extensive brownish-yellow granular and patchy staining in the model group, which was significantly improved by H-BKA treatment (Figure 4G). Collectively, these findings suggest that BKA can mitigate liver injury induced by APAP.

### 3.3. Untargeted Metabolomics Profiling

**Figure 5:**
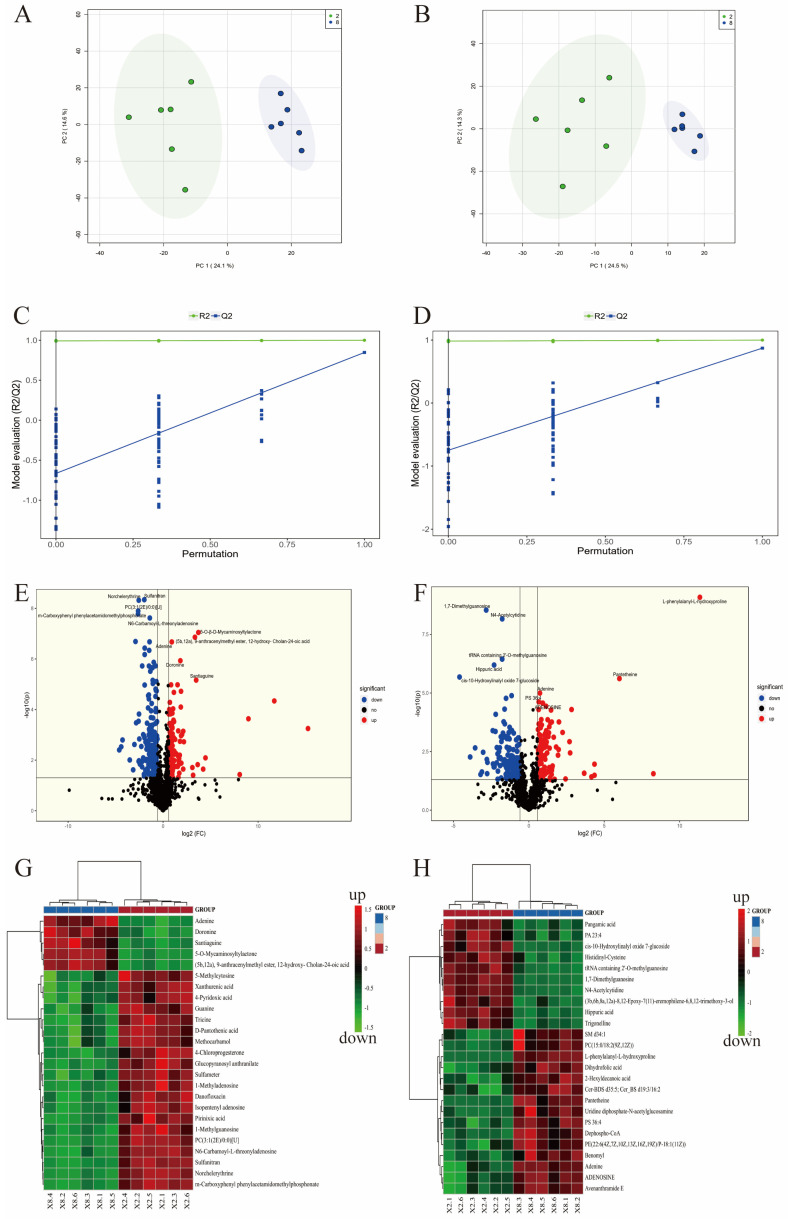
PCA analysis shows the separation between the BKA high-dose and model groups (green represents the model group, blue represents the BKA high-dose group) (**A**,**B**). PLS-DA permutation test plots for the BKA high-dose vs. model group (**C**,**D**). Volcano plots display the positive and negative differential metabolites between the BKA high-dose and the model groups (**E**,**F**). Heatmaps for the positive and negative differential metabolites between the BKA high-dose and the model group (**G**,**H**).

To further clarify the pharmacological mechanisms of BKA in mitigating APAP-induced ALI, the present study performed an untargeted metabolomics analysis on liver samples, focusing on the metabolomics results of the model and high-dose groups. Our findings revealed a distinct separation trend in the liver metabolic profiles between the two groups in the principal component analysis (PCA) score plot (Figure 5A). The partial least squares discriminant analysis (PLS-DA) score plot (Figure 5B) also demonstrated a significant separation between the two groups. The permutation test plot of the PLS-DA model showed that both Q2 and R2 values exceeded 0.5, confirming a well-fitted model. Differential metabolites were identified using the PLS-DA model and *t*-tests, with varying significance levels in the variable importance in projection (VIP) values and *p*-values. The screening threshold for differential metabolites was set at *p* < 0.05 and VIP > 1.0.

In the positive ion mode, compared with the model group, the high-dose group exhibited 148 upregulated and 186 downregulated differential metabolites (Figure 5E). A heatmap of the metabolites revealed key differential metabolites, including xanthurenic acid, guanosine, *D*-pantothenic acid, and tricine (Figure 5G). In the negative ion mode, the high-dose group exhibited 110 upregulated and 153 downregulated differential metabolites compared to the model group (Figure 5F). A heatmap of the metabolites revealed key differential metabolites, including trigonelline, pantetheine, adenosine, dephospho-CoA, uridine diphosphate-*N*-acetylgl, and dihydrofolic acid (Figure 5H).

**Figure 6:**
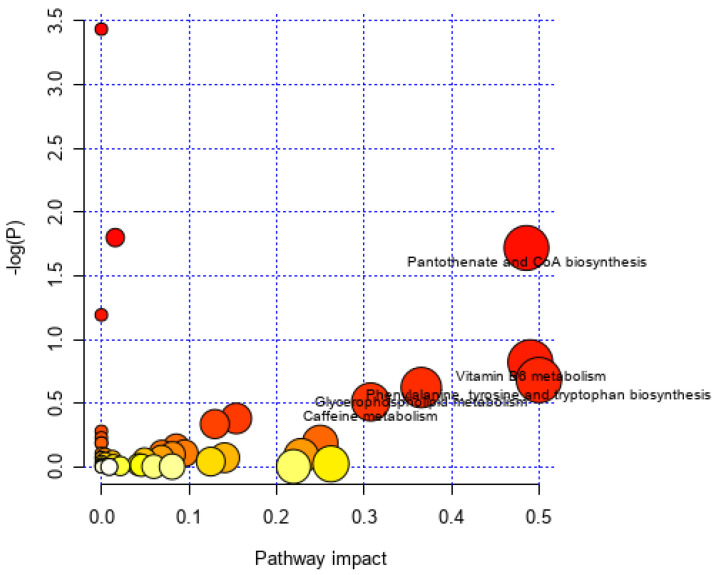
Metabolomics KEGG analysis. KEGG enrichment map comparing the high-dose group with the model group.

Further KEGG enrichment analysis (Figure 6) indicated that the high-dose group primarily affected the following pathways compared with the model group: pantothenate and CoA biosynthesis, vitamin B6 metabolism, glycerophospholipid metabolism, phenylalanine, tyrosine, and tryptophan biosynthesis, and caffeine metabolism. Some of the related metabolites and pathways are closely associated with oxidative stress. Therefore, this study further investigated how BKA alleviates APAP-induced ALI by modulating oxidative stress.

### 3.4. Effect of BKA on Oxidative Stress Protein Expression in the Liver of Mice Treated with APAP

**Figure 7:**
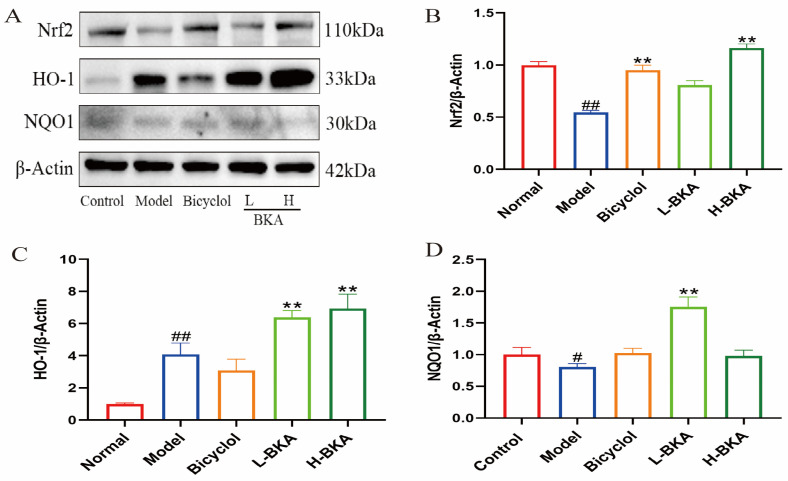
(**A**) Western blot analysis of Nrf2, HO-1, and NQO1 proteins. (**B**–**D**) Trends in protein expression: Nrf2, HO-1, and NQO1. ^#^ *p* < 0.05 vs. control; ^##^ *p* < 0.01 vs. control; ** *p* < 0.01 vs. Model.

Western blot analysis further confirmed the molecular mechanism underlying BKA’s protective effect against APAP-induced ALI. Our results demonstrated that the expression of the HO-1 proteins was downregulated considerably following APAP-induced liver injury (Figure 7). However, high-dose BKA (100 mg/kg) administration could modulate the expression of these proteins and alleviate the expression of oxidative stress-related proteins activated or inhibited by APAP.

### 3.5. Molecular Docking Results

**Figure 8:**
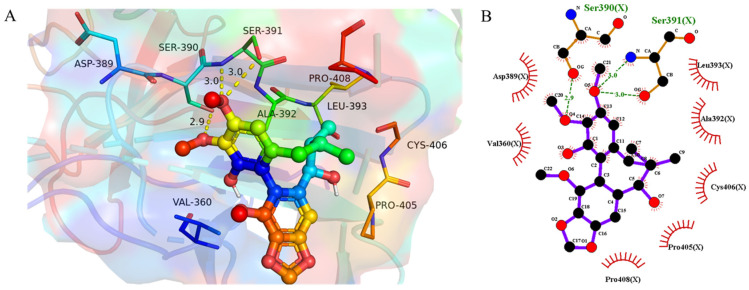
Molecular docking analysis of BKA-Keap1 (PDB code: 4IF1). (**A**) Predicted binding interactions of BKA with key amino acid residues in Keap1. (**B**) 2D interaction diagram of BKA with Keap1.

To explore the possible binding mode of BKA to the Keap1 Kelch domain, molecular docking was performed using AutoDock software. From Figure 8, it was clearly seen that the CH_3_O group at C-2 and Ser390 formed a hydrogen bond. Additionally, the CH_3_O group at C-3 binds to Ser391 through hydrogen bonding. Moreover, BKA engaged with Asp389, Val360, Leu393, Ala392, Cys406, Pro405, and Pro408 via hydrophobic interactions. The docking binding energy was −5.6 kcal/mol, indicating a strong affinity between BKA and the Keap1 Kelch domain. Therefore, BKA could directly block the binding site of Nrf2 on Keap1 and prevent the binding of Nrf2, resulting in its activation.

## 4. Discussion

APAP, a widely available over-the-counter analgesic and antipyretic, is consumed globally in large quantities. While safe at therapeutic doses (≤4 g/day), its overdose precipitates drug-induced liver injury (DILI) and constitutes a formidable public health challenge [21]. In fact, APAP hepatotoxicity has become the leading cause of acute liver failure worldwide. In Europe and the United States, approximately 2000 cases of acute liver failure occur annually, nearly half of which are attributable to inappropriate APAP use [22]. The underlying mechanism involves the cytochrome P450-mediated bioactivation of APAP to the reactive metabolite NAPQI. At therapeutic levels, NAPQI is efficiently detoxified via conjugation with GSH to yield non-toxic mercapturates that are subsequently excreted. However, supratherapeutic doses exhaust hepatic GSH reserves, allowing NAPQI to bind covalently to cellular proteins, provoking oxidative stress and excessive ROS generation [23]. Currently, NAC remains the only FDA-approved antidote for APAP-induced hepatotoxicity, but its narrow therapeutic window and adverse effects restrict its clinical utility [24]. Therefore, it is urgently desirable to develop effective drugs to prevent APAP-induced liver injury.

*Kadsura coccinea* belongs to the genus *Kadsura* in the family Schisandraceae. Phytochemical studies have shown that it is rich in lignans and triterpenoids. The lignans in *K. coccinea* exhibit excellent hepatoprotective effects [25,26]. This study isolated a dibenzocyclooctadiene lignan, BKA, from *K. coccinea* fruits. Its structural characteristics were determined using spectroscopic techniques. Additionally, the single-crystal X-ray diffraction of BKA was reported for the first time (Figure 3). Previous researchers have found that dibenzocyclooctadiene lignans possess the activity to alleviate APAP-induced hepatotoxicity [17]. However, the specific mechanism remains unclear. Therefore, the current study designed in vivo experiments to explore how BKA alleviates APAP-induced liver injury.

Aspartate aminotransferase (AST) and alanine aminotransferase (ALT) are key biochemical markers for evaluating liver function. When hepatocytes are damaged or necrotic, intracellular AST and ALT are released into the bloodstream, resulting in elevated serum levels of these enzymes [27]. Cytochrome P450 2E1 (CYP2E1) is the primary enzyme responsible for the metabolism of APAP to NAPQI. NAPQI covalently binds to hepatocyte proteins, triggering oxidative stress and mitochondrial dysfunction, ultimately leading to hepatocyte necrosis [28]. Studies have shown that CYP2E1 inducers (such as alcohol) can increase APAP-induced hepatotoxicity, while CYP2E1 inhibitors can mitigate the damage [29]. GSH is a detoxifying agent that counteracts NAPQI-induced hepatotoxicity. Excessive NAPQI depletes GSH stores, leading to oxidative stress, mitochondrial dysfunction, and DNA damage, ultimately resulting in necrotic and apoptotic cell death [30]. In this study, the model group showed elevated serum levels of AST and ALT in mice. High-dose BKA was able to counteract this effect, indicating that BKA can alleviate APAP-induced liver injury. Meanwhile, the model group exhibited a decrease in hepatic GSH content and increased CYP2E1 expression. High-dose BKA alleviated APAP-induced liver injury by regulating GSH levels and CYP2E1 expression.

Omics technologies, such as metabolomics, are powerful tools for investigating biological systems. They enable the identification of diverse metabolites and their pathway associations, thereby elucidating pathophysiological mechanisms [31]. When the liver is damaged, the most crucial metabolic organ in the human body exhibits significant metabolic dysregulation [32]. Recent research indicates that many researchers are leveraging metabolomics techniques to study liver-related diseases. For instance, metabolomics combined with network pharmacology has been employed to uncover the protective effects of astragaloside IV against alcoholic liver disease [33]. The mechanisms of rice protein in alleviating alcoholic liver disease have been explored through liver metabolomics and gut microbiota analysis [34]. By integrating metabolomic and transcriptomic analyses, the potential mechanisms of polyphenol-enriched extracts from raspberry fruits in mitigating non-alcoholic fatty liver disease have been investigated [35]. Similarly, this study utilized metabolomics to explore the impact of BKA on APAP-induced liver metabolic disorders. PCA results indicated significant metabolic differences between the high-dose BKA and APAP groups. This suggests that APAP alters the liver metabolic profile in mice, while BKA can counteract APAP-induced metabolic dysregulation. Different metabolites between the APAP group and the high-dose BKA group were identified through comparative analysis. Notably, among the differential metabolites, xanthurenic acid [36], guanosine [37], trigonelline [38], and adenosine [39] can alleviate oxidative stress to some extent. Subsequent KEGG pathway enrichment analysis showed that key metabolic pathways, including Vitamin B6 metabolism [40], phenylalanine, tyrosine, and tryptophan biosynthesis [41], and caffeine metabolism [42], are closely associated with oxidative stress.

**Figure 9:**
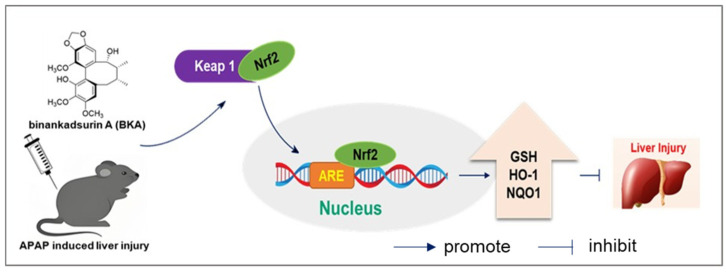
A mechanism for BKA ameliorates APAP-induced liver injury through inhibiting oxidative stress by Keap1/Nrf2/HO-1 pathways.

The Nrf2 pathway plays a crucial role in defending against APAP-induced oxidative stress [43,44]. Under normal physiological conditions, the redox-sensitive transcription factor Nrf2 is sequestered in the cytoplasm by interacting with Keap1. However, during oxidative stress, Nrf2 dissociates from Keap1. It translocates into the nucleus, where it binds to the ARE and activates the expression of downstream antioxidant enzymes, such as HO-1 and NQO1, thereby exerting antioxidant effects [45]. Given the significance of the Nrf2 pathway in APAP-induced liver injury, we investigated the impact of BKA on this pathway. The experimental results demonstrated that APAP significantly suppressed the expression of Nrf2 and NQO1 proteins. In contrast, BKA treatment upregulated the expression of Nrf2 and NQO1 and promoted the expression of the antioxidant enzyme HO-1. The present study also performed molecular docking analysis of BKA with Keap1 to further explore the underlying mechanism. The results indicated a binding energy of −5.6 kcal/mol, suggesting a strong binding affinity of BKA for Keap1. These findings suggest a strong binding affinity between BKA and the Keap1 Kelch domain. Therefore, BKA directly blocks the binding site of Nrf2 with Keap1 and prevents the binding of Nrf2, resulting in its activation, thereby countering the oxidative stress associated with APAP-induced liver injury (Figure 9).

To date, no clinical trials of BKA have been conducted. Future directions should prioritize optimizing its pharmacokinetic profile (solubility, absorption, metabolism) via structural modification or nanodelivery systems, validating in vivo efficacy in disease-specific models, and developing scalable total synthesis to address low isolation yields, laying the groundwork for potential clinical translation.

## 5. Conclusions

In this study, a dibenzocyclooctadiene lignan, BKA, was isolated from the fruits of *K. coccinea*. The structure of BKA was characterized using techniques such as NMR and X-ray single-crystal diffraction. By integrating experimental verification and non-targeted metabolomics methods, this study explored the protective effect of BKA against APAP-induced acute liver injury. Nontargeted metabolomics analysis revealed that BKA counteracted APAP-induced liver metabolic disorders by regulating metabolites and pathways closely related to oxidative stress. Subsequent mechanism verification demonstrated that BKA could modulate the Nrf2 pathway to alleviate APAP-induced oxidative stress. In summary, our results indicate that BKA mitigates APAP-induced liver injury by regulating pathways associated with oxidative stress. However, this is only a preliminary exploration of pharmacodynamics and mechanisms, with certain limitations. In the future, detailed mechanism research will be carried out.

Despite promising bioactivity, BKA suffers from low isolation yields and critical translational challenges: poor aqueous solubility, inadequate absorption, rapid metabolism, and limited tissue distribution hinder its administration. Additionally, its complex chemical structure poses substantial hurdles to total synthesis, which remains a key bottleneck for scalable production and further clinical development.

## Figures and Tables

**Table 1:** ^1^H NMR (600 MHz), ^13^C NMR (150 MHz) data of BKA in CDCl_3_ and key ^1^H–^1^H COSY and HMBC correlations.

NO.	*δ_H_* (*J* in Hz)	*δ_C_*	^1^H-^1^H COSY	HMBC
1	-	147.3	-	-
2	-	133.8	-	-
3	-	151.3	-	-
4	6.41 (s)	107.4	-	H-4 → C-3, C-5, C-6, C-16
5	-	133.8	-	-
6	2.62 (m); 2.63 (m)	38.8	H-6 ↔ H-7	H-6 → C-4, C-5, C-7, C-8, C-16, C-18
7	2.07 (m)	34.9	H-7 ↔ H-6, H-8	H-7 → C-6, C-8, C-9, C-18
8	1.91 (m)	43.0	H8 ↔ H-7, H-9	H-8 → C-7, C-9, C-17, C-18
9	4.63 (s)	83.6	H-9 ↔ H-8	H-9 → C-7, C-8, C-11, C-15, C-17
10	-	138.8	-	-
11	6.36 (s)	102.8	-	H-11 → C-9, C-12, C-13, C-15
12	-	148.9	-	-
13	-	135.7	-	-
14	-	141.4	-	-
15	-	118.3	-	-
16	-	115.1	-	-
17	0.93 (d, 7.4)	19.7	H-17 ↔ H-8	H_3_-17 → C-8
18	1.16 (d, 7.2)	15.4	H-18 ↔ H-7	H_3_-18 → C-7
OCH_2_O	5.95 (s); 5.97 (s)	101.2	-	H-19 → C-10, C-12
2-OCH_3_	3.87 (s)	59.8	-	H_3_-2 → C-2
3-OCH_3_	3.90 (s)	61.1	-	H_3_-3 → C-3
14-OCH_3_	3.88 (s)	55.7	-	H_3_-14 → C-14

## Data Availability

The original contributions presented in this study are included in the article/Supplementary Material. Further inquiries can be directed to the corresponding authors.

## Supplementary Materials

The following supporting information can be downloaded at: https://www.mdpi.com/article/10.3390/nu18030403/s1, Figure S1 shows the HR-ESI-MS spectrum of BKA. Figures S2–S7 reveal NMR data. Figures S8–S11 original protein gel.

## Abbreviations

The following abbreviations are used in this manuscript:

ALI	Acute liver injury
ALT	Alanine aminotransferase
AST	Aspartate aminotransferase
APAP	Acetaminophen
APAP-ADs	APAP protein adducts
ARE	Antioxidant response elements
BKA	Binankadsurin A
DILI	Drug-induced liver injury
HO-1	Heme oxygenase-1
GSH	Glutathione
NAC	N-acetylcysteine
NAPQI	N-Acetyl-p-benzoquinone imine
NQO1	NAD (P) H quinone oxidoreductase 1
NMR	Nuclear Magnetic Resonance
Nrf2	Nuclear factor E2-related factor 2
PCA	Principal component analysis
PDB	Protein Data Bank
PLS-DA	Partial least squares discriminant analysis
ROS	Reactive oxygen species
VIP	Variable importance in projection
