# Supplementary material for: Binankadsurin A from Kadsura coccinea Fruits Ameliorates Acetaminophen-Induced Liver Injury Through Inhibiting Oxidative Stress by Keap1/Nrf2/HO-1 Pathway
Source: Nutrients. 2026 Jan 26;18(3):403. doi: 10.3390/nu18030403 (PMC12899676; doi:10.3390/nu18030403)
Supplement: Supplementary file 1 [file nutrients-18-00403-s001.zip › nutrients-4031286-supplementary.pdf]

Supplementary Materials

Binankadsurin A from *Kadsura coccinea* Fruits Ameliorates
Acetaminophen-Induced Liver Injury Through Inhibiting
Oxidative Stress by Keap1/Nrf2/HO-1 Pathway

**Guy Paulin M. Kemayou [1,2,†], Yashi Wang [1,†], Muhammad Aamer [1], Chuanle Li [1], Shiqi Liu [1], Huanghe Yu [1],
Caiyun Peng [1], Simeon F. Kouam [2], Bin Li [1], Wei Wang [1,*] and Yupei Yang [1,*]**

[1] TCM and Ethnomedicine Innovation & Development International Laboratory, School of Pharmacy, Hunan University of Chinese Medicine, Changsha 410208, China; guybeni93@gmail.com (G.P.M.K.); 18173724730@163.com (Y.W.); m.aamer196267@gmail.com (M.A.); 14794431027@139.com (C.L.); shiqiliu670@163.com (S.L.); yhh@hnucm.edu.cn (H.Y.); caiyunpeng@hnucm.edu.cn (C.P.); libin@hnucm.edu.cn (B.L.)

[2] Department of Chemistry, Higher Teacher Training College, University of Yaounde I, Yaounde P.O. Box 47, Cameroon; kfogue@yahoo.com

* Correspondence: wangwei402@hotmail.com (W.W.); yangyupei24@163.com (Y.Y.)

† These authors contributed equally to this work.

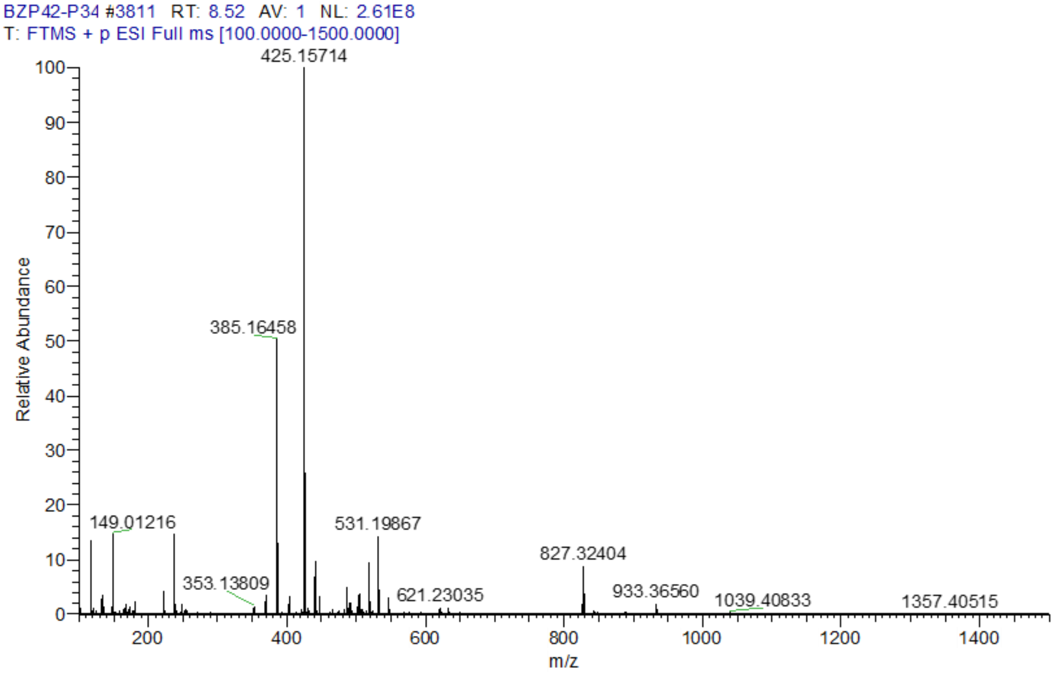

Figure S1 HRESIMS spectrum of **BKA**

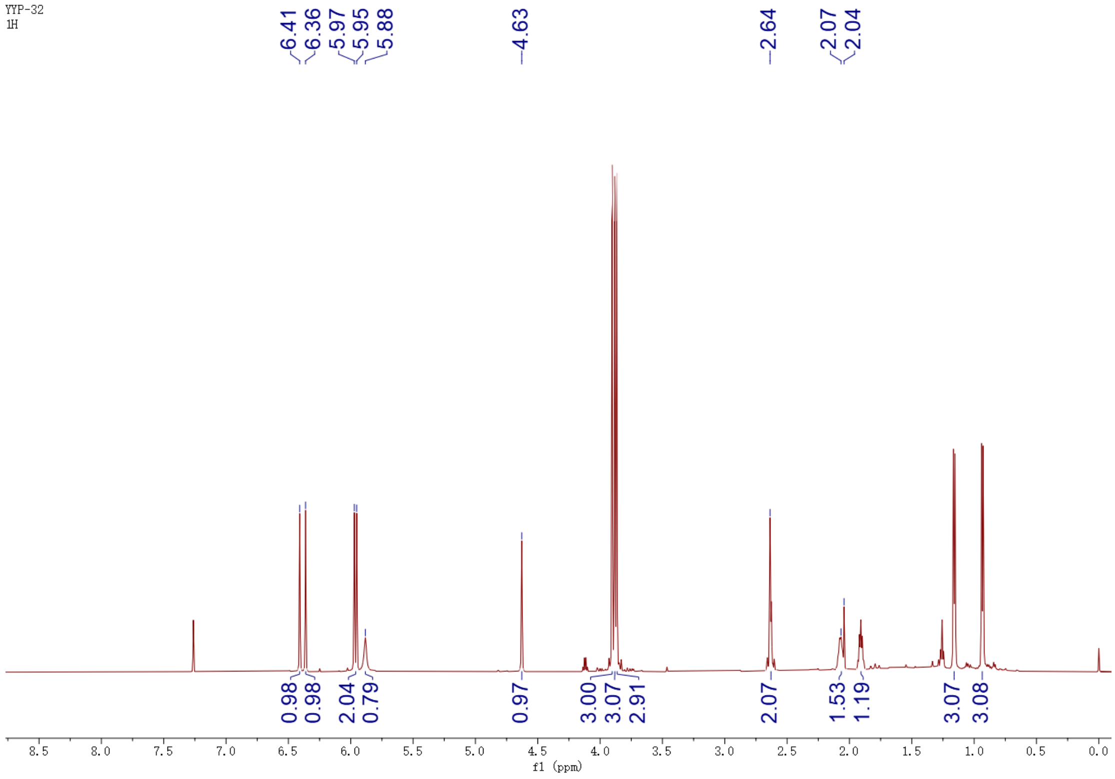

Figure S2 ^{1}H NMR (600 MHz, $CDCl_3$) spectrum of **BKA**

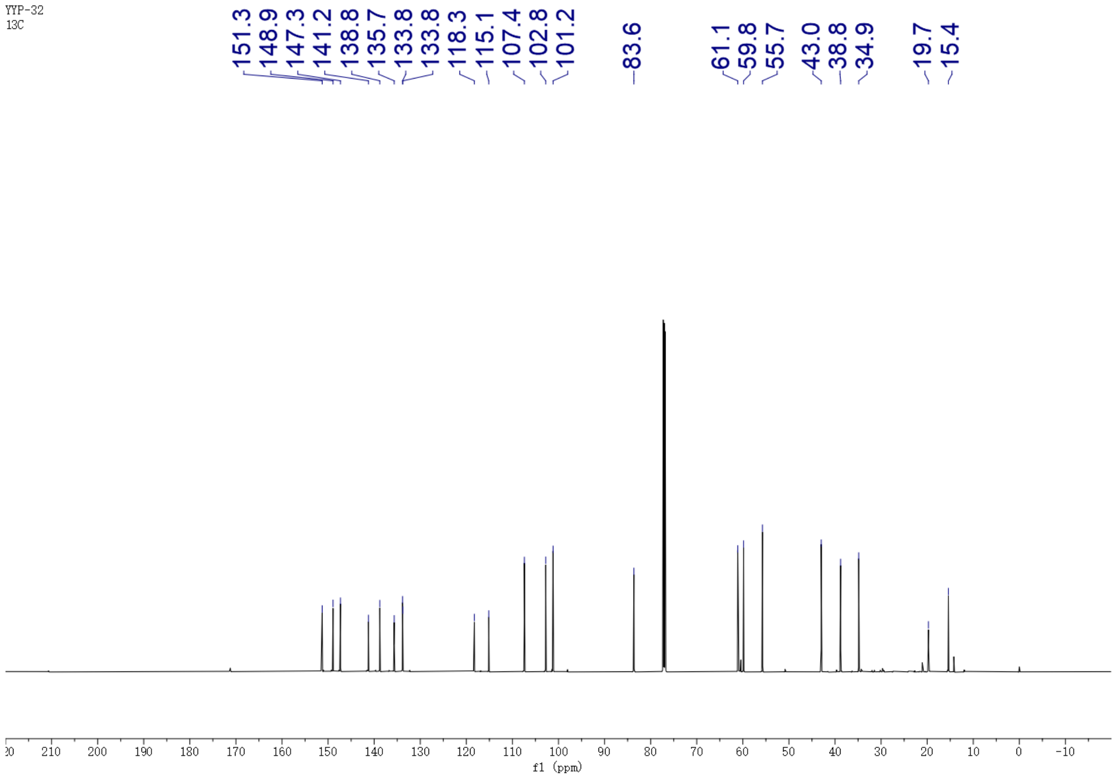

Figure S3 ^{13}C NMR (150 MHz, $CDCl_3$) spectrum of **BKA**

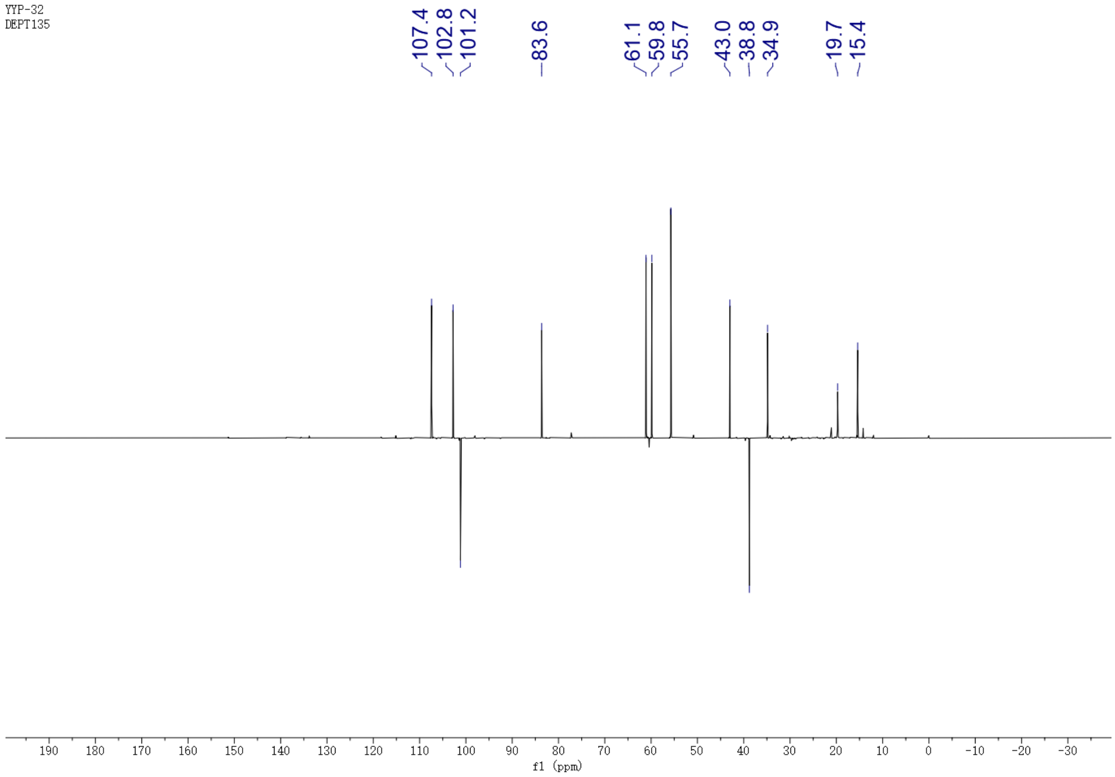

Figure S4 DEPT 135°NMR (150 MHz, $CDCl_3$) spectrum of **BKA**

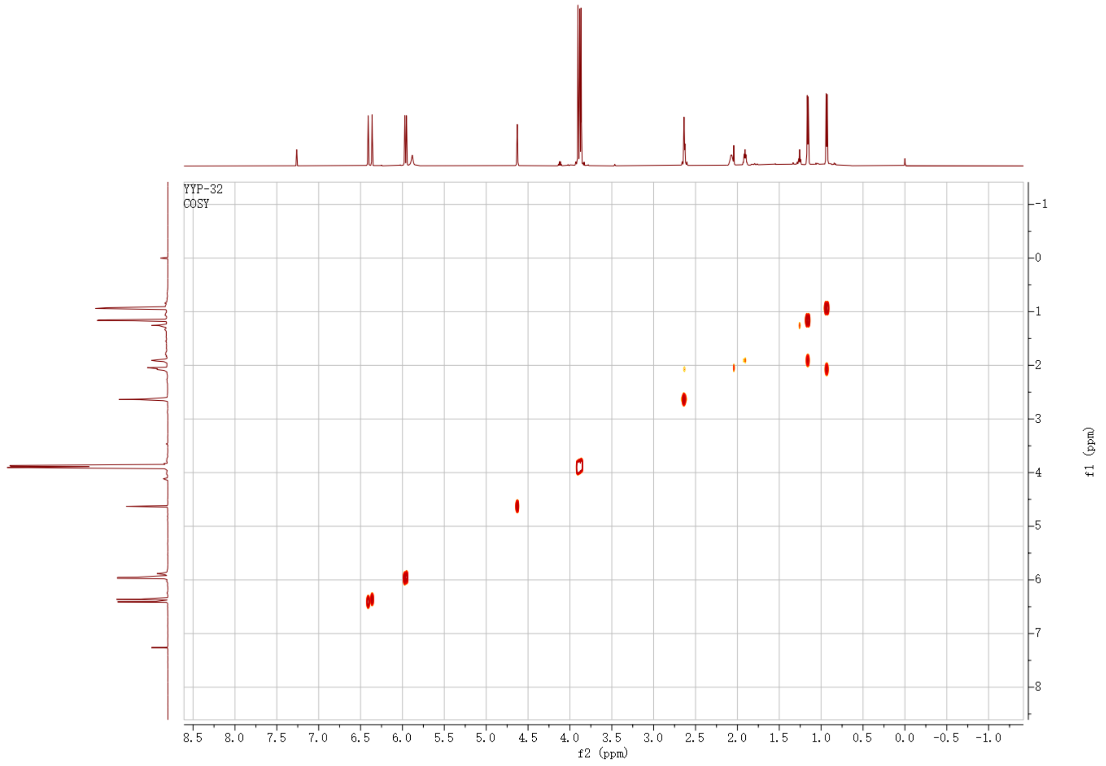

Figure S5 ^{1}H-^{1}H COSY NMR (600 MHz, $CDCl_3$) spectrum of **BKA**

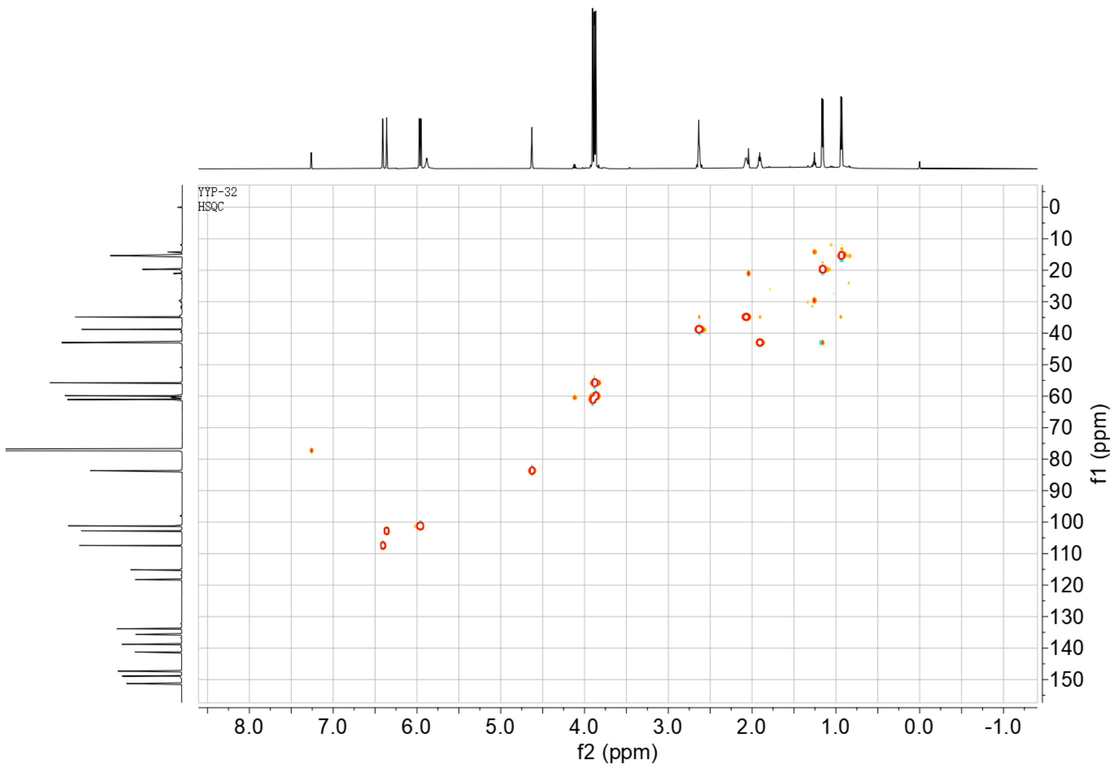

Figure S6 HSQC NMR (600 MHz, $CDCl_3$) spectrum of **BKA**

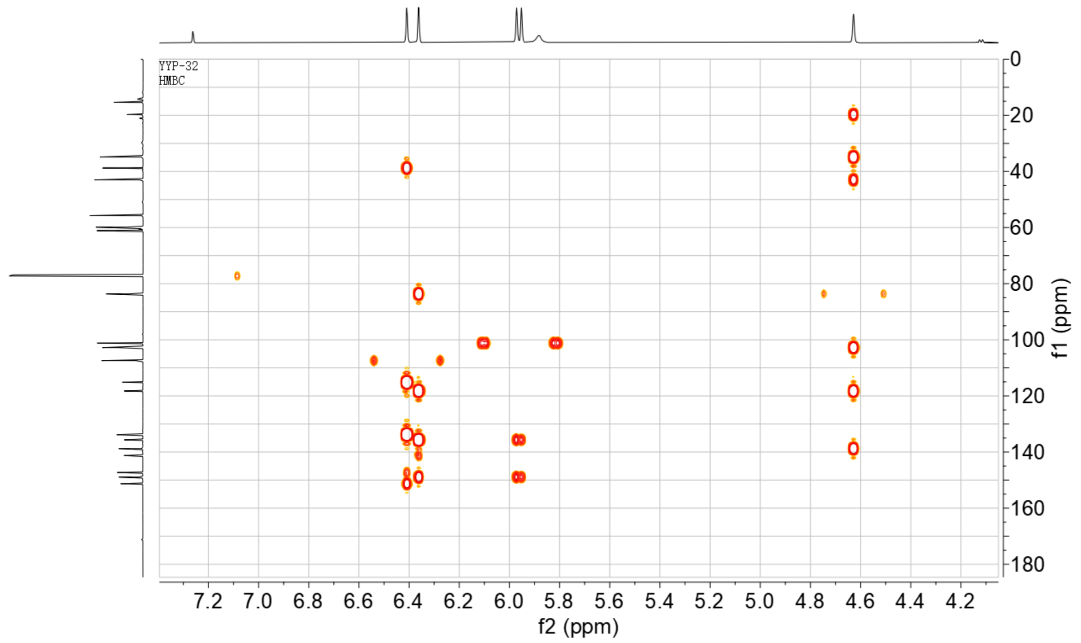

Figure S7 HMBC NMR (600 MHz, $CDCl_3$) spectrum of **BKA**

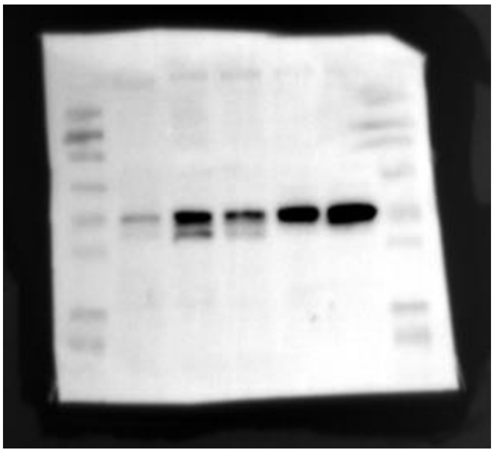

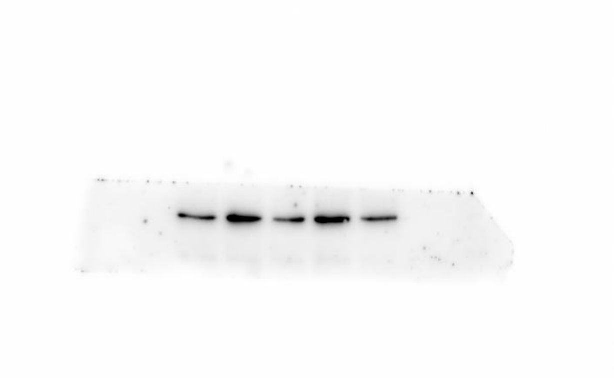

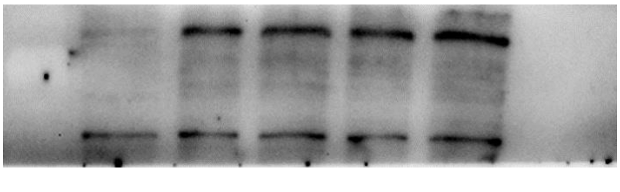

Figure S8 Original gel of HO-1 (33 kDa)

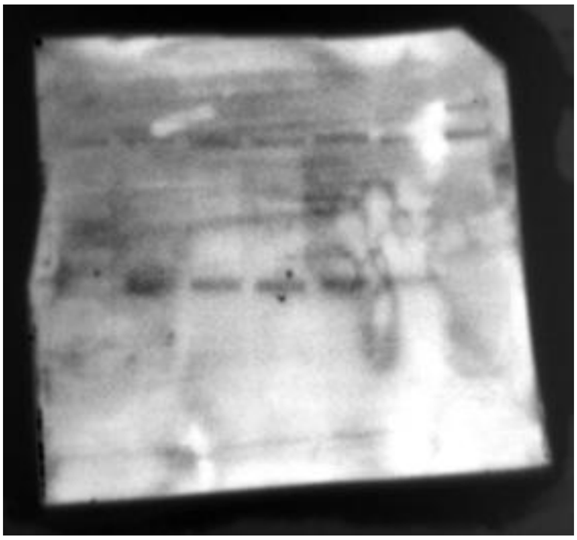

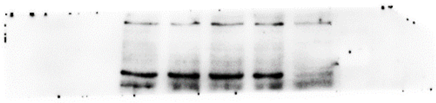

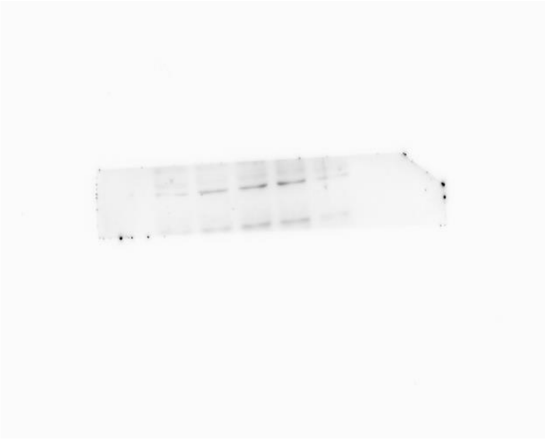

Figure S9 Original gel of NQO1 (30 kDa)

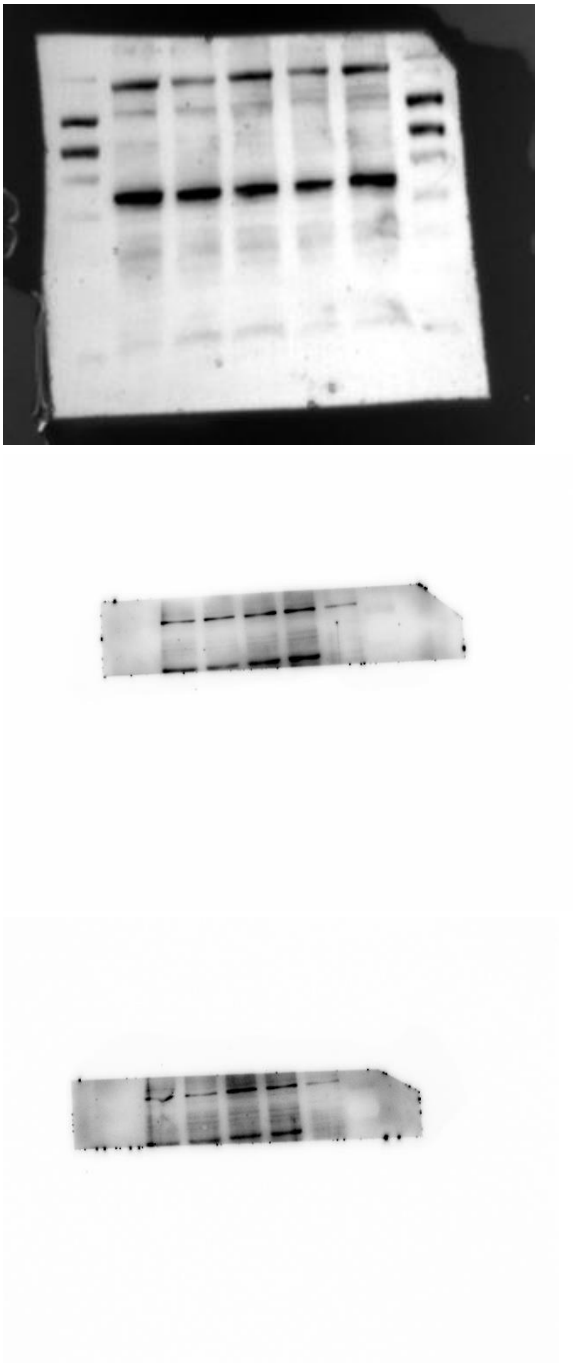

Figure S10 Original gel of Nrf2 (110 kDa)

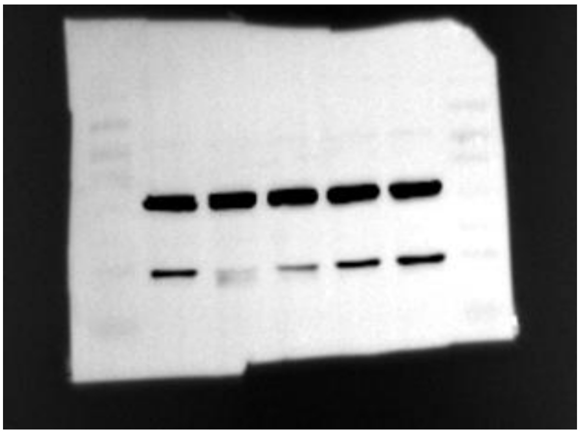

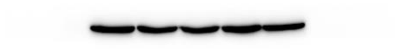

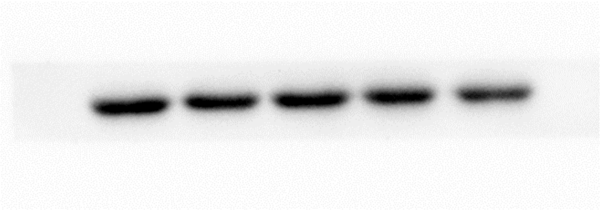

Figure S11 Original gel of β-Actin (41 kDa)
